# 3,3-Dimethyl-*cis*-9a,13a-diphenyl-2,3,9a,11,12,13a-hexa­hydro-1*H*-benzo[*h*][1,4]dioxino[2′,3′:5,6][1,4]dioxino[2,3-*f*]chromene

**DOI:** 10.1107/S1600536813023660

**Published:** 2013-08-31

**Authors:** Bauer O. Bernardes, Aurelio B. Buarque Ferreira, James L. Wardell, Solange M. S. V. Wardell, José C. Netto-Ferreira, Edward R. T. Tiekink

**Affiliations:** a Departamento de Química, Universidade Federal Rural do Rio de Janeiro, 23851-970 Seropédica, RJ, Brazil; b Instituto de Tecnologia em Fármacos–Farmanguinhos, Fundação Oswaldo Cruz, 21041-250 Rio de Janeiro, RJ, Brazil; c Department of Chemistry, University of Aberdeen, Old Aberdeen AB24 3UE, Scotland; d CHEMSOL, 1 Harcourt Road, Aberdeen AB15 5NY, Scotland; e Department of Chemistry, University of Malaya, 50603 Kuala Lumpur, Malaysia

## Abstract

In the title di­hydro­dioxin, C_31_H_28_O_5_, the dioxane ring has a chair conformation, whereas each of the pyran and dioxine rings has an envelope conformation with methyl­ene and quaternary C atoms, respectively, being the flap atoms. The phenyl rings are *cis* and form a dihedral angle of 82.11 (10)°. The molecular structure is stabilized by C—H⋯O contacts. In the crystal packing, supra­molecular layers parallel to (101) are sustained by C—H⋯π inter­actions.

## Related literature

For the biological activity of lapachol and its isomers, see: de Almeida (2009 ▶); Ferreira *et al.* (2010 ▶); Medeiros *et al.* (2010 ▶); Neves-Pinto *et al.* (2002 ▶). For reactions of the quinone O atoms in lapachol, see: da Silva *et al.* (2011 ▶); Ferreira *et al.* (2006 ▶); Neves-Pinto *et al.* (2002 ▶). For the preparation of di­hydro­dioxins, see: Schönberg & Mustafa (1944 ▶), and for their DNA photo-cleavage, see: Mack *et al.* (2004 ▶). For the synthesis, see: Summerbell & Berger (1959 ▶). For the crystal structure of β-lapachone, see: Cunha-Filho *et al.* (2006 ▶).
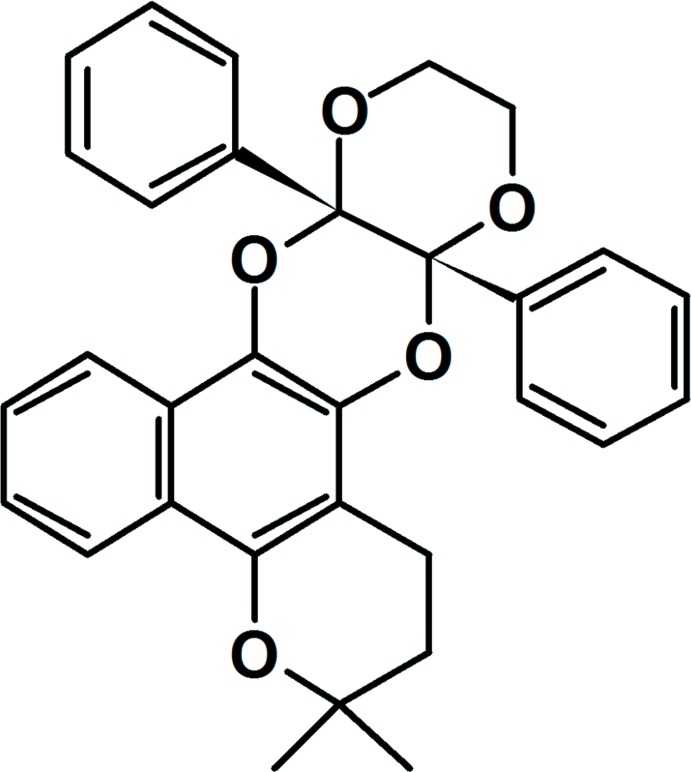


## Experimental

### 

#### Crystal data

C_31_H_28_O_5_
*M* *_r_* = 480.53
Monoclinic, 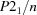

*a* = 15.1335 (6) Å
*b* = 9.6048 (2) Å
*c* = 16.9739 (6) Å
β = 97.384 (1)°
*V* = 2446.77 (14) Å^3^
*Z* = 4
Mo *K*α radiation
μ = 0.09 mm^−1^
*T* = 120 K
0.36 × 0.28 × 0.07 mm

#### Data collection

Bruker-Nonius Roper CCD camera on a κ-goniostat diffractometer
Absorption correction: multi-scan (*SADABS*; Sheldrick, 2003 ▶) *T*_min_ = 0.831, *T*_max_ = 1.000
23103 measured reflections
5549 independent reflections
3390 reflections with *I* > 2σ(*I*)
*R*_int_ = 0.059

#### Refinement

*R*[*F* ^2^ > 2σ(*F* ^2^)] = 0.050
*wR*(*F* ^2^) = 0.139
*S* = 1.02
5549 reflections
355 parameters
H-atom parameters constrained
Δρ_max_ = 0.25 e Å^−3^
Δρ_min_ = −0.29 e Å^−3^

### 

Data collection: *COLLECT* (Hooft, 1998 ▶); cell refinement: *DENZO* (Otwinowski & Minor, 1997 ▶) and *COLLECT*; data reduction: *DENZO* and *COLLECT*; program(s) used to solve structure: *SHELXS97* (Sheldrick, 2008 ▶); program(s) used to refine structure: *SHELXL97* (Sheldrick, 2008 ▶); molecular graphics: *ORTEP-3 for Windows* (Farrugia, 2012 ▶) and *DIAMOND* (Brandenburg, 2006 ▶); software used to prepare material for publication: *publCIF* (Westrip, 2010 ▶).

## Supplementary Material

Crystal structure: contains datablock(s) general, I. DOI: 10.1107/S1600536813023660/hg5343sup1.cif

Structure factors: contains datablock(s) I. DOI: 10.1107/S1600536813023660/hg5343Isup2.hkl

Additional supplementary materials:  crystallographic information; 3D view; checkCIF report

## Figures and Tables

**Table 1:** Hydrogen-bond geometry (Å, °) *Cg*1 is the centroid of the C4*A*,C5,C6,C6*A*,C10*A*,C10*B* benzene ring.

*D*—H⋯*A*	*D*—H	H⋯*A*	*D*⋯*A*	*D*—H⋯*A*
C18—H18⋯O3	0.95	2.36	3.001 (3)	124
C22—H22⋯O4	0.95	2.32	2.683 (3)	102
C24—H24⋯O4	0.95	2.44	3.071 (2)	124
C8—H8⋯*Cg*1^i^	0.95	2.65	3.3134 (19)	128
C15—H15*A*⋯*Cg*1^ii^	0.99	2.39	3.336 (2)	161

Symmetry codes: (i) 
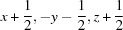
; (ii) 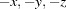.

**Fig. 1.**
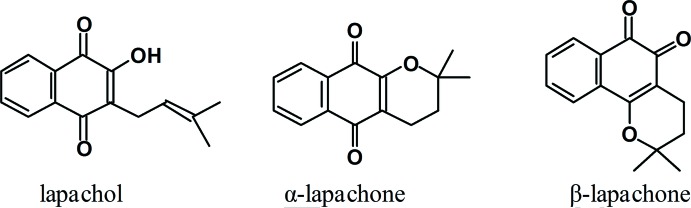
Chemical structures of lapachol, α-lapachone and β-lapachone

**Fig. 2.**
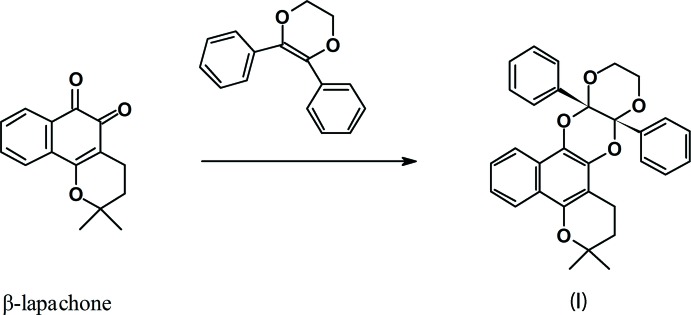
Reaction scheme for the synthesis of the title compound, (I).

**Fig. 3.**
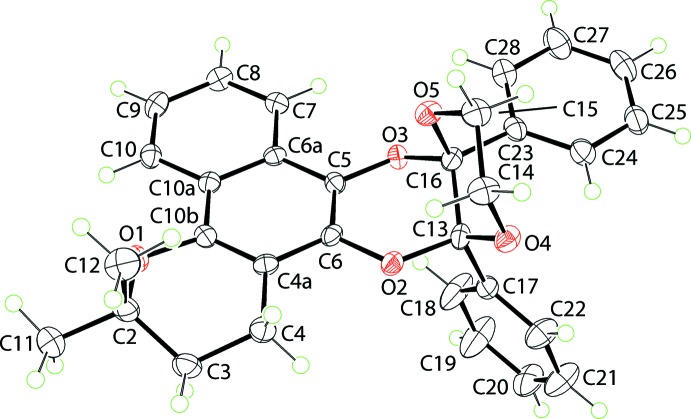
The molecular structure of (I) showing the atom-labelling scheme and displacement ellipsoids at the 50% probability level.

**Fig. 4.**
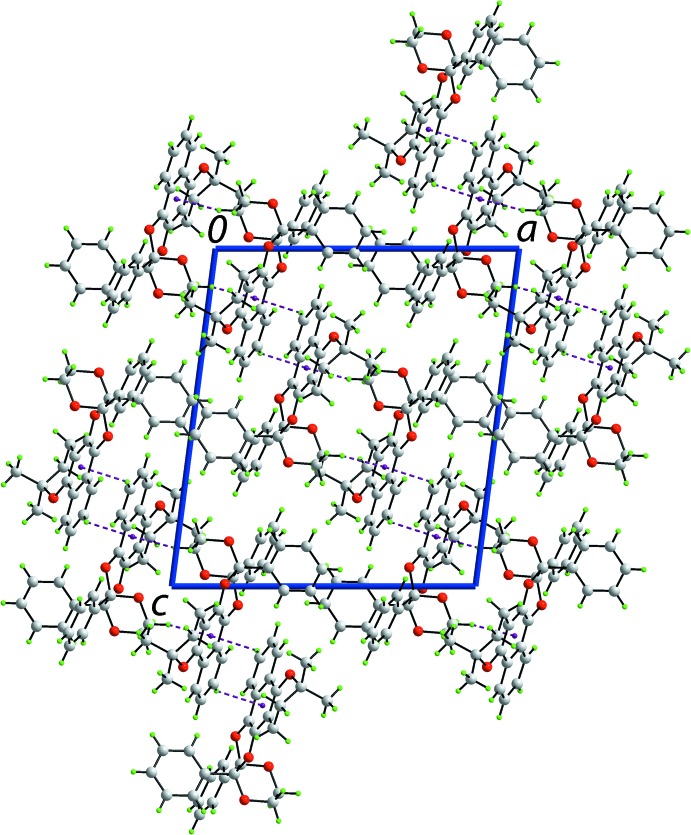
A view in projection down the b axis of the unit-cell contents of (I). The C—H···π interactions are shown as purple dashed lines respectively.

## supplementary crystallographic information

### 1. Comment

Isomeric, lapachol, 2-hydroxy-3-(3-methyl-2-butenyl)-1,4-naphthoquinone, β-lapachone, 2,2-dimethyl-3,4-dihydro-2*H*-benzo[*h*]chromene-5,6-dione, and α-lapachone, 2,2-dimethyl-3,4-dihydro-2*H*-benzo[*g*]chromene-5,10-dione, Fig. 1, are found in the wood of trees of the genus, *Tabebuia* (family *Bignoniaceae*), distributed throughout Central and South America. Since their discovery at the end of the 19th century, lapachol and its isomers have attracted much attention due to their biological activities (de Almeida, 2009; Ferreira *et al.*, 2010). Studies have revealed the effectiveness of these compounds and their derivatives as anti-cancer and anti-neoplastic (de Almeida, 2009), anti-fungal (Medeiros *et al.*, 2010) and anti-Trypanosoma cruzi agents (Neves-Pinto *et al.*, 2002), among other activities.

The quinone O atoms in lapachol and the lapachones are active sites and reactions at these sites have led to various derivatives, including oximes (da Silva *et al.*, 2011), α-diazocarbonyls (Ferreira *et al.*, 2006), phenazines (Neves-Pinto *et al.*, 2002) and as we report here, a dihydrodioxin, (I), which was obtained by photoaddition of β-lapachone to 5,6-diphenyl-2,3-dihydro-1,4-dioxine, Fig. 2. Dihydrodioxins, most readily formed by a photochemical reaction between *ortho*-quinones and alkenes (Schönberg & Mustafa, 1944), are able to perform efficient DNA photo-cleavage (Mack *et al.*, 2004). The crystal structure of β-lapachone has been reported (Cunha-Filho *et al.*, 2006).

In (I), Fig. 3, the pyran ring approximates an envelope conformation with the C3 atom being the flap atom. The dioxine ring also has an envelope conformation where the C16 atom is the flap. With respect to this ring, the C17- and C23-bound phenyl rings are in axial and equatorial positions, respectively, and make a dihedral angle of 82.11 (10)° with each other. The orientation of these rings is such to facilitate the formation of intramolecular C—H···O interactions, Table 1. Finally, a chair conformation is found for the dioxane ring.

The major feature of the crystal packing is the formation of supramolecular layers parallel to (1 0 1) and sustained by C—H···π interactions, Table 1. These stack with no specific intermolecular interactions between them, Fig. 4.

### 2. Experimental

β-Lapachone (0.242 g, 1 mmol) was added to a solution of 2,3-diphenyl-1,4-diox-2-ene (0.476 g, 2 mmol) in benzene (20 ml) (Summerbell & Berger, 1959). The solution was deaerated using oxygen-free nitrogen and irradiated using a medium-pressure Hg lamp (500 W; irradiation time = 15 h). The solvent was removed under reduced pressure to leave a residue, to which was added methanol (20 ml). This mixture was filtered under reduced pressure, the colourless solid was collected, and recrystallized from ethanol; *M*.pt: 482–484 K, yield 69%. Colourless blocks were obtained by slow evaporation of a 1:9 dichloromethane:acetonitrile solution at room temperature. UV (acetonitrile, λ_max_. (ε) - nm, *L*.mol^-1^.cm^-1^): 212 (3.9x10^4^), 245.5 (3.08x10^4^), 317 (5.8x10^3^). IR (KBr) (cm^-1^): 3065.4, 2972.7, 2935.9, 1646.2, 1586.0, 1495.4, 1450.1, 1413.3, 1389.4, 1326.2, 1264.9, 1240.4, 1180.6, 1160.1, 1105.0, 1068.7, 1042.0, 1018.4, 953.3, 914.2, 854.2, 765.1, 725.9. GC—MS *m*/*z* (abundance): 480 (<1%), 238 (11%), 214 (1%), 199 (1%), 181 (1%), 159 (1%), 130 (1%), 105 (100%), 77 (17%), 51 (2%). HRMS: *m/z* 480.2020 (theoretical 480.2036) ^1^H NMR (CDCl_3_) δ (p.p.m.): 8.16 (1*H*, m); 8.12 (1*H*, m); 7.77–7.68 (4*H*, m); 7.44 (1*H*, dt, J = 7.02 and 1.36 Hz); 7.32 (1*H*, dt, J = 6.20 and 1.36 Hz); 7.24–7.18 (6*H*, m); 4.34–4.14 (2*H*, m); 3.96–3.89 (2*H*, m); 3.02–2.76 (2*H*, m), 1.87 (2*H*, J = 6.48 and 1.62 Hz); 1.42 (3*H*, s); 1.38 (3*H*, s). ^13^C NMR (CDCl_3_) δ (p.p.m.): 17.39; 26.63; 26.82; 32.09; 61.41; 61.78; 74.03; 94.43; 95.10; 106.6; 119.75; 121.54; 123.27; 123.99. 125.57; 127.27; 127.67; 128.52; 134.46. 137.46; 137.79; 144.10.

### 3. Refinement

The C-bound H atoms were geometrically placed (C—H = 0.95–0.99 Å) and refined as riding with *U*_iso_(H) = 1.2–1.5*U*_eq_(C).

### Crystal data

**Table:**

C_31_H_28_O_5_	*F*(000) = 1016
*M**_r_* = 480.53	*D*_x_ = 1.304 Mg m^−^^3^
Monoclinic, *P*2_1_/*n*	Mo *K*α radiation, λ = 0.71073 Å
Hall symbol: -P 2yn	Cell parameters from 5547 reflections
*a* = 15.1335 (6) Å	θ = 2.9–27.5°
*b* = 9.6048 (2) Å	µ = 0.09 mm^−^^1^
*c* = 16.9739 (6) Å	*T* = 120 K
β = 97.384 (1)°	Slab, colourless
*V* = 2446.77 (14) Å^3^	0.36 × 0.28 × 0.07 mm
*Z* = 4	

### Data collection

**Table:**

Bruker-Nonius Roper CCD camera on a κ-goniostat diffractometer	5549 independent reflections
Radiation source: Bruker–Nonius FR591 rotating anode	3390 reflections with *I* > 2σ(*I*)
Graphite monochromator	*R*_int_ = 0.059
Detector resolution: 9.091 pixels mm^-1^	θ_max_ = 27.4°, θ_min_ = 3.2°
φ & ω scans	*h* = −15→19
Absorption correction: multi-scan (*SADABS*; Sheldrick, 2003)	*k* = −12→12
*T*_min_ = 0.831, *T*_max_ = 1.000	*l* = −21→21
23103 measured reflections	

### Refinement

**Table:**

Refinement on *F*^2^	Primary atom site location: structure-invariant direct methods
Least-squares matrix: full	Secondary atom site location: difference Fourier map
*R*[*F*^2^ > 2σ(*F*^2^)] = 0.050	Hydrogen site location: inferred from neighbouring sites
*wR*(*F*^2^) = 0.139	H-atom parameters constrained
*S* = 1.02	*w* = 1/[σ^2^(*F*_o_^2^) + (0.0726*P*)^2^] where *P* = (*F*_o_^2^ + 2*F*_c_^2^)/3
5549 reflections	(Δ/σ)_max_ < 0.001
355 parameters	Δρ_max_ = 0.25 e Å^−^^3^
0 restraints	Δρ_min_ = −0.29 e Å^−^^3^

### Special details

**Table:**

Geometry. All s.u.'s (except the s.u. in the dihedral angle between two l.s. planes) are estimated using the full covariance matrix. The cell s.u.'s are taken into account individually in the estimation of s.u.'s in distances, angles and torsion angles; correlations between s.u.'s in cell parameters are only used when they are defined by crystal symmetry. An approximate (isotropic) treatment of cell s.u.'s is used for estimating s.u.'s involving l.s. planes.
Refinement. Refinement of *F*^2^ against ALL reflections. The weighted *R*-factor *wR* and goodness of fit *S* are based on *F*^2^, conventional *R*-factors *R* are based on *F*, with *F* set to zero for negative *F*^2^. The threshold expression of *F*^2^ > 2σ(*F*^2^) is used only for calculating *R*-factors(gt) *etc*. and is not relevant to the choice of reflections for refinement. *R*-factors based on *F*^2^ are statistically about twice as large as those based on *F*, and *R*- factors based on ALL data will be even larger.

### Fractional atomic coordinates and isotropic or equivalent isotropic displacement parameters (Å^2^)

**Table:**

	*x*	*y*	*z*	*U*_iso_*/*U*_eq_	
O1	0.07265 (9)	0.34146 (11)	0.23877 (7)	0.0227 (3)	
O2	0.17388 (8)	0.21646 (11)	−0.00687 (7)	0.0212 (3)	
O3	0.22585 (8)	−0.04598 (11)	0.05583 (7)	0.0197 (3)	
O4	0.16943 (9)	0.13692 (12)	−0.13294 (7)	0.0241 (3)	
O5	0.10415 (8)	−0.05976 (11)	−0.03375 (7)	0.0220 (3)	
C2	0.03141 (13)	0.46851 (17)	0.20263 (11)	0.0249 (4)	
C3	0.09216 (14)	0.52936 (17)	0.14656 (11)	0.0272 (5)	
H3A	0.1507	0.5521	0.1769	0.031 (5)*	
H3B	0.0658	0.6168	0.1232	0.030 (5)*	
C4	0.10551 (14)	0.42770 (17)	0.08004 (11)	0.0246 (4)	
H4A	0.0510	0.4243	0.0410	0.025 (5)*	
H4B	0.1555	0.4595	0.0522	0.024 (5)*	
C4A	0.12557 (12)	0.28463 (16)	0.11431 (10)	0.0188 (4)	
C5	0.18803 (12)	0.05363 (16)	0.10082 (10)	0.0172 (4)	
C6	0.16391 (12)	0.18128 (17)	0.06989 (10)	0.0184 (4)	
C6A	0.17505 (12)	0.01886 (16)	0.17947 (10)	0.0171 (4)	
C7	0.19548 (12)	−0.11445 (17)	0.21269 (10)	0.0200 (4)	
H7	0.2207	−0.1834	0.1823	0.013 (4)*	
C8	0.17912 (12)	−0.14461 (18)	0.28843 (10)	0.0231 (4)	
H8	0.1916	−0.2351	0.3095	0.019 (5)*	
C9	0.14399 (13)	−0.04266 (18)	0.33515 (11)	0.0253 (5)	
H9	0.1342	−0.0639	0.3880	0.036 (6)*	
C10	0.12390 (13)	0.08757 (17)	0.30443 (10)	0.0216 (4)	
H10	0.1010	0.1562	0.3366	0.031 (5)*	
C10A	0.13687 (12)	0.12094 (17)	0.22567 (10)	0.0181 (4)	
C10B	0.11111 (12)	0.25221 (17)	0.19031 (10)	0.0184 (4)	
C11	0.02511 (16)	0.56118 (19)	0.27419 (12)	0.0355 (5)	
H11A	0.0849	0.5773	0.3023	0.046 (7)*	
H11B	−0.0118	0.5156	0.3100	0.035 (6)*	
H11C	−0.0018	0.6504	0.2564	0.046 (6)*	
C12	−0.06075 (14)	0.4327 (2)	0.16053 (13)	0.0343 (5)	
H12A	−0.0552	0.3644	0.1185	0.045 (6)*	
H12B	−0.0893	0.5172	0.1371	0.040 (6)*	
H12C	−0.0971	0.3933	0.1989	0.033 (6)*	
C13	0.21410 (13)	0.11661 (18)	−0.05551 (10)	0.0207 (4)	
C14	0.07502 (13)	0.10801 (18)	−0.13920 (11)	0.0267 (5)	
H14A	0.0477	0.1202	−0.1950	0.026 (5)*	
H14B	0.0464	0.1745	−0.1058	0.034 (5)*	
C15	0.05956 (14)	−0.03797 (18)	−0.11270 (11)	0.0256 (5)	
H15A	−0.0051	−0.0544	−0.1136	0.031 (5)*	
H15B	0.0823	−0.1049	−0.1496	0.022 (5)*	
C16	0.19741 (12)	−0.03490 (17)	−0.02661 (10)	0.0193 (4)	
C17	0.31092 (13)	0.15683 (17)	−0.05503 (10)	0.0220 (4)	
C18	0.37148 (16)	0.1396 (3)	0.01208 (12)	0.0485 (7)	
H18	0.3530	0.0973	0.0578	0.069 (8)*	
C19	0.45920 (16)	0.1832 (3)	0.01376 (13)	0.0561 (7)	
H19	0.5001	0.1697	0.0605	0.082 (9)*	
C20	0.48716 (16)	0.2452 (2)	−0.05084 (13)	0.0407 (6)	
H20	0.5471	0.2755	−0.0494	0.053 (7)*	
C21	0.42738 (15)	0.2634 (2)	−0.11828 (14)	0.0427 (6)	
H21	0.4464	0.3058	−0.1638	0.048 (6)*	
C22	0.33982 (14)	0.2206 (2)	−0.12029 (13)	0.0341 (5)	
H22	0.2991	0.2351	−0.1670	0.050 (7)*	
C23	0.24471 (13)	−0.15056 (17)	−0.06662 (10)	0.0216 (4)	
C24	0.27317 (14)	−0.13653 (19)	−0.14065 (11)	0.0285 (5)	
H24	0.2670	−0.0496	−0.1674	0.042 (6)*	
C25	0.31066 (14)	−0.2488 (2)	−0.17594 (12)	0.0321 (5)	
H25	0.3301	−0.2380	−0.2266	0.045 (6)*	
C26	0.31977 (15)	−0.3756 (2)	−0.13795 (12)	0.0345 (5)	
H26	0.3456	−0.4520	−0.1622	0.038 (6)*	
C27	0.29106 (15)	−0.3912 (2)	−0.06409 (12)	0.0351 (5)	
H27	0.2971	−0.4786	−0.0377	0.037 (6)*	
C28	0.25370 (13)	−0.28000 (18)	−0.02881 (12)	0.0282 (5)	
H28	0.2339	−0.2916	0.0217	0.031 (5)*	

### Atomic displacement parameters (Å^2^)

**Table:**

	*U*^11^	*U*^22^	*U*^33^	*U*^12^	*U*^13^	*U*^23^
O1	0.0249 (8)	0.0204 (6)	0.0237 (7)	0.0053 (5)	0.0061 (6)	−0.0014 (5)
O2	0.0252 (8)	0.0203 (6)	0.0191 (7)	0.0007 (5)	0.0068 (6)	0.0029 (5)
O3	0.0209 (8)	0.0211 (6)	0.0173 (6)	0.0028 (5)	0.0035 (5)	−0.0014 (5)
O4	0.0229 (8)	0.0300 (7)	0.0189 (7)	−0.0040 (5)	0.0003 (6)	0.0026 (5)
O5	0.0152 (7)	0.0267 (6)	0.0234 (7)	−0.0026 (5)	−0.0002 (6)	0.0017 (5)
C2	0.0244 (12)	0.0183 (9)	0.0323 (11)	0.0036 (8)	0.0051 (9)	−0.0006 (8)
C3	0.0301 (12)	0.0177 (9)	0.0344 (11)	0.0019 (8)	0.0058 (10)	0.0010 (8)
C4	0.0273 (12)	0.0212 (9)	0.0260 (10)	0.0019 (8)	0.0056 (9)	0.0024 (8)
C4A	0.0154 (10)	0.0187 (9)	0.0220 (10)	−0.0021 (7)	0.0007 (8)	0.0016 (7)
C5	0.0141 (10)	0.0186 (9)	0.0191 (9)	0.0001 (7)	0.0024 (8)	−0.0031 (7)
C6	0.0156 (10)	0.0229 (9)	0.0169 (9)	−0.0050 (7)	0.0029 (8)	0.0002 (7)
C6A	0.0132 (10)	0.0186 (8)	0.0189 (9)	−0.0028 (7)	0.0001 (8)	0.0009 (7)
C7	0.0170 (11)	0.0191 (9)	0.0233 (10)	0.0008 (7)	0.0003 (8)	−0.0004 (7)
C8	0.0186 (11)	0.0246 (10)	0.0251 (10)	0.0022 (8)	−0.0007 (8)	0.0064 (8)
C9	0.0229 (12)	0.0327 (11)	0.0208 (10)	0.0005 (8)	0.0044 (9)	0.0051 (8)
C10	0.0194 (11)	0.0250 (9)	0.0210 (10)	0.0006 (8)	0.0044 (8)	−0.0020 (8)
C10A	0.0138 (10)	0.0213 (9)	0.0190 (9)	−0.0019 (7)	0.0010 (8)	−0.0007 (7)
C10B	0.0135 (10)	0.0201 (8)	0.0213 (10)	−0.0004 (7)	0.0011 (8)	−0.0031 (7)
C11	0.0412 (15)	0.0277 (11)	0.0398 (13)	0.0035 (10)	0.0137 (11)	−0.0045 (9)
C12	0.0246 (13)	0.0333 (11)	0.0444 (13)	0.0052 (9)	0.0025 (10)	0.0053 (10)
C13	0.0203 (11)	0.0269 (9)	0.0154 (9)	−0.0005 (8)	0.0048 (8)	−0.0017 (7)
C14	0.0211 (12)	0.0299 (10)	0.0272 (11)	−0.0021 (8)	−0.0042 (9)	0.0037 (8)
C15	0.0205 (12)	0.0304 (10)	0.0237 (10)	−0.0015 (8)	−0.0049 (9)	0.0001 (8)
C16	0.0164 (11)	0.0257 (9)	0.0156 (9)	−0.0034 (7)	0.0012 (8)	0.0000 (7)
C17	0.0204 (11)	0.0238 (9)	0.0225 (10)	−0.0036 (8)	0.0057 (9)	−0.0035 (7)
C18	0.0318 (15)	0.0891 (18)	0.0237 (12)	−0.0267 (13)	0.0000 (11)	0.0119 (11)
C19	0.0288 (15)	0.110 (2)	0.0274 (13)	−0.0285 (14)	−0.0049 (11)	0.0131 (13)
C20	0.0234 (13)	0.0611 (14)	0.0387 (13)	−0.0159 (11)	0.0079 (11)	−0.0002 (11)
C21	0.0281 (14)	0.0603 (14)	0.0405 (14)	−0.0078 (11)	0.0072 (11)	0.0179 (11)
C22	0.0230 (12)	0.0445 (12)	0.0344 (12)	−0.0028 (9)	0.0027 (10)	0.0121 (9)
C23	0.0171 (11)	0.0250 (10)	0.0222 (10)	−0.0012 (8)	0.0005 (8)	−0.0057 (7)
C24	0.0295 (13)	0.0298 (10)	0.0262 (11)	−0.0041 (9)	0.0033 (9)	−0.0061 (8)
C25	0.0295 (13)	0.0384 (12)	0.0295 (12)	−0.0050 (9)	0.0081 (10)	−0.0128 (9)
C26	0.0334 (13)	0.0337 (11)	0.0358 (12)	0.0060 (9)	0.0021 (10)	−0.0154 (9)
C27	0.0420 (15)	0.0278 (11)	0.0347 (12)	0.0109 (9)	0.0014 (11)	−0.0049 (9)
C28	0.0290 (13)	0.0303 (11)	0.0250 (11)	0.0032 (8)	0.0028 (9)	−0.0037 (8)

### Geometric parameters (Å, º)

**Table:**

O1—C10B	1.369 (2)	C11—H11B	0.9800
O1—C2	1.468 (2)	C11—H11C	0.9800
O2—C6	1.373 (2)	C12—H12A	0.9800
O2—C13	1.449 (2)	C12—H12B	0.9800
O3—C5	1.3923 (19)	C12—H12C	0.9800
O3—C16	1.414 (2)	C13—C17	1.514 (3)
O4—C13	1.412 (2)	C13—C16	1.567 (2)
O4—C14	1.446 (2)	C14—C15	1.500 (2)
O5—C16	1.421 (2)	C14—H14A	0.9900
O5—C15	1.436 (2)	C14—H14B	0.9900
C2—C11	1.519 (3)	C15—H15A	0.9900
C2—C12	1.523 (3)	C15—H15B	0.9900
C2—C3	1.522 (3)	C16—C23	1.527 (2)
C3—C4	1.526 (2)	C17—C18	1.377 (3)
C3—H3A	0.9900	C17—C22	1.385 (3)
C3—H3B	0.9900	C18—C19	1.389 (3)
C4—C4A	1.508 (2)	C18—H18	0.9500
C4—H4A	0.9900	C19—C20	1.362 (3)
C4—H4B	0.9900	C19—H19	0.9500
C4A—C10B	1.372 (2)	C20—C21	1.376 (3)
C4A—C6	1.415 (2)	C20—H20	0.9500
C5—C6	1.365 (2)	C21—C22	1.384 (3)
C5—C6A	1.414 (2)	C21—H21	0.9500
C6A—C7	1.417 (2)	C22—H22	0.9500
C6A—C10A	1.424 (2)	C23—C24	1.386 (3)
C7—C8	1.371 (2)	C23—C28	1.398 (3)
C7—H7	0.9500	C24—C25	1.390 (3)
C8—C9	1.407 (2)	C24—H24	0.9500
C8—H8	0.9500	C25—C26	1.376 (3)
C9—C10	1.374 (2)	C25—H25	0.9500
C9—H9	0.9500	C26—C27	1.387 (3)
C10—C10A	1.413 (2)	C26—H26	0.9500
C10—H10	0.9500	C27—C28	1.381 (3)
C10A—C10B	1.429 (2)	C27—H27	0.9500
C11—H11A	0.9800	C28—H28	0.9500
			
C10B—O1—C2	117.40 (13)	H12A—C12—H12C	109.5
C6—O2—C13	118.89 (12)	H12B—C12—H12C	109.5
C5—O3—C16	113.37 (12)	O4—C13—O2	104.65 (13)
C13—O4—C14	113.10 (13)	O4—C13—C17	108.48 (14)
C16—O5—C15	113.43 (13)	O2—C13—C17	107.76 (13)
O1—C2—C11	102.70 (15)	O4—C13—C16	110.04 (13)
O1—C2—C12	108.83 (14)	O2—C13—C16	109.86 (13)
C11—C2—C12	111.10 (17)	C17—C13—C16	115.48 (15)
O1—C2—C3	108.77 (15)	O4—C14—C15	110.38 (15)
C11—C2—C3	112.34 (15)	O4—C14—H14A	109.6
C12—C2—C3	112.56 (17)	C15—C14—H14A	109.6
C2—C3—C4	111.41 (15)	O4—C14—H14B	109.6
C2—C3—H3A	109.3	C15—C14—H14B	109.6
C4—C3—H3A	109.3	H14A—C14—H14B	108.1
C2—C3—H3B	109.3	O5—C15—C14	110.08 (14)
C4—C3—H3B	109.3	O5—C15—H15A	109.6
H3A—C3—H3B	108.0	C14—C15—H15A	109.6
C4A—C4—C3	109.69 (15)	O5—C15—H15B	109.6
C4A—C4—H4A	109.7	C14—C15—H15B	109.6
C3—C4—H4A	109.7	H15A—C15—H15B	108.2
C4A—C4—H4B	109.7	O3—C16—O5	104.15 (13)
C3—C4—H4B	109.7	O3—C16—C23	106.55 (13)
H4A—C4—H4B	108.2	O5—C16—C23	110.91 (13)
C10B—C4A—C6	117.89 (15)	O3—C16—C13	109.81 (13)
C10B—C4A—C4	121.41 (15)	O5—C16—C13	109.13 (14)
C6—C4A—C4	120.64 (15)	C23—C16—C13	115.64 (14)
C6—C5—O3	120.98 (15)	C18—C17—C22	118.01 (18)
C6—C5—C6A	120.83 (15)	C18—C17—C13	120.96 (16)
O3—C5—C6A	118.18 (14)	C22—C17—C13	120.89 (17)
C5—C6—O2	121.97 (15)	C17—C18—C19	120.9 (2)
C5—C6—C4A	122.20 (15)	C17—C18—H18	119.6
O2—C6—C4A	115.82 (14)	C19—C18—H18	119.6
C5—C6A—C7	122.64 (15)	C20—C19—C18	120.7 (2)
C5—C6A—C10A	118.22 (15)	C20—C19—H19	119.7
C7—C6A—C10A	119.10 (15)	C18—C19—H19	119.7
C8—C7—C6A	120.50 (16)	C19—C20—C21	119.1 (2)
C8—C7—H7	119.7	C19—C20—H20	120.5
C6A—C7—H7	119.7	C21—C20—H20	120.5
C7—C8—C9	120.56 (16)	C20—C21—C22	120.5 (2)
C7—C8—H8	119.7	C20—C21—H21	119.7
C9—C8—H8	119.7	C22—C21—H21	119.7
C10—C9—C8	120.06 (16)	C21—C22—C17	120.8 (2)
C10—C9—H9	120.0	C21—C22—H22	119.6
C8—C9—H9	120.0	C17—C22—H22	119.6
C9—C10—C10A	120.97 (16)	C24—C23—C28	118.65 (16)
C9—C10—H10	119.5	C24—C23—C16	123.39 (16)
C10A—C10—H10	119.5	C28—C23—C16	117.79 (16)
C10—C10A—C6A	118.73 (15)	C23—C24—C25	120.46 (18)
C10—C10A—C10B	122.27 (15)	C23—C24—H24	119.8
C6A—C10A—C10B	118.98 (15)	C25—C24—H24	119.8
O1—C10B—C4A	123.68 (15)	C26—C25—C24	120.42 (19)
O1—C10B—C10A	114.48 (14)	C26—C25—H25	119.8
C4A—C10B—C10A	121.83 (15)	C24—C25—H25	119.8
C2—C11—H11A	109.5	C25—C26—C27	119.69 (18)
C2—C11—H11B	109.5	C25—C26—H26	120.2
H11A—C11—H11B	109.5	C27—C26—H26	120.2
C2—C11—H11C	109.5	C28—C27—C26	120.10 (19)
H11A—C11—H11C	109.5	C28—C27—H27	119.9
H11B—C11—H11C	109.5	C26—C27—H27	119.9
C2—C12—H12A	109.5	C27—C28—C23	120.67 (18)
C2—C12—H12B	109.5	C27—C28—H28	119.7
H12A—C12—H12B	109.5	C23—C28—H28	119.7
C2—C12—H12C	109.5		
			
C10B—O1—C2—C11	−161.60 (15)	C6—O2—C13—O4	145.39 (14)
C10B—O1—C2—C12	80.58 (19)	C6—O2—C13—C17	−99.29 (17)
C10B—O1—C2—C3	−42.4 (2)	C6—O2—C13—C16	27.3 (2)
O1—C2—C3—C4	60.8 (2)	C13—O4—C14—C15	−56.94 (18)
C11—C2—C3—C4	173.82 (16)	C16—O5—C15—C14	−57.79 (19)
C12—C2—C3—C4	−59.9 (2)	O4—C14—C15—O5	55.3 (2)
C2—C3—C4—C4A	−46.2 (2)	C5—O3—C16—O5	−62.68 (16)
C3—C4—C4A—C10B	14.3 (2)	C5—O3—C16—C23	−179.99 (13)
C3—C4—C4A—C6	−162.79 (17)	C5—O3—C16—C13	54.07 (18)
C16—O3—C5—C6	−29.5 (2)	C15—O5—C16—O3	173.41 (12)
C16—O3—C5—C6A	150.33 (15)	C15—O5—C16—C23	−72.34 (16)
O3—C5—C6—O2	1.4 (3)	C15—O5—C16—C13	56.18 (16)
C6A—C5—C6—O2	−178.46 (16)	O4—C13—C16—O3	−167.72 (13)
O3—C5—C6—C4A	−179.82 (16)	O2—C13—C16—O3	−53.02 (18)
C6A—C5—C6—C4A	0.3 (3)	C17—C13—C16—O3	69.09 (18)
C13—O2—C6—C5	−2.2 (2)	O4—C13—C16—O5	−54.14 (17)
C13—O2—C6—C4A	178.95 (15)	O2—C13—C16—O5	60.56 (17)
C10B—C4A—C6—C5	−1.3 (3)	C17—C13—C16—O5	−177.33 (13)
C4—C4A—C6—C5	175.85 (17)	O4—C13—C16—C23	71.70 (19)
C10B—C4A—C6—O2	177.52 (16)	O2—C13—C16—C23	−173.60 (14)
C4—C4A—C6—O2	−5.3 (2)	C17—C13—C16—C23	−51.5 (2)
C6—C5—C6A—C7	177.14 (17)	O4—C13—C17—C18	−176.17 (18)
O3—C5—C6A—C7	−2.7 (3)	O2—C13—C17—C18	71.1 (2)
C6—C5—C6A—C10A	−0.5 (3)	C16—C13—C17—C18	−52.2 (2)
O3—C5—C6A—C10A	179.63 (15)	O4—C13—C17—C22	8.2 (2)
C5—C6A—C7—C8	−177.93 (18)	O2—C13—C17—C22	−104.54 (18)
C10A—C6A—C7—C8	−0.3 (3)	C16—C13—C17—C22	132.24 (18)
C6A—C7—C8—C9	−1.7 (3)	C22—C17—C18—C19	−0.9 (3)
C7—C8—C9—C10	1.5 (3)	C13—C17—C18—C19	−176.6 (2)
C8—C9—C10—C10A	0.8 (3)	C17—C18—C19—C20	0.6 (4)
C9—C10—C10A—C6A	−2.8 (3)	C18—C19—C20—C21	−0.4 (4)
C9—C10—C10A—C10B	175.73 (17)	C19—C20—C21—C22	0.6 (4)
C5—C6A—C10A—C10	−179.76 (16)	C20—C21—C22—C17	−0.9 (3)
C7—C6A—C10A—C10	2.5 (3)	C18—C17—C22—C21	1.1 (3)
C5—C6A—C10A—C10B	1.7 (2)	C13—C17—C22—C21	176.80 (19)
C7—C6A—C10A—C10B	−176.06 (16)	O3—C16—C23—C24	−145.17 (17)
C2—O1—C10B—C4A	10.7 (2)	O5—C16—C23—C24	102.1 (2)
C2—O1—C10B—C10A	−170.26 (15)	C13—C16—C23—C24	−22.8 (2)
C6—C4A—C10B—O1	−178.42 (16)	O3—C16—C23—C28	39.6 (2)
C4—C4A—C10B—O1	4.4 (3)	O5—C16—C23—C28	−73.1 (2)
C6—C4A—C10B—C10A	2.6 (3)	C13—C16—C23—C28	161.93 (16)
C4—C4A—C10B—C10A	−174.60 (16)	C28—C23—C24—C25	−0.6 (3)
C10—C10A—C10B—O1	−0.4 (2)	C16—C23—C24—C25	−175.84 (18)
C6A—C10A—C10B—O1	178.11 (15)	C23—C24—C25—C26	0.2 (3)
C10—C10A—C10B—C4A	178.70 (17)	C24—C25—C26—C27	0.2 (3)
C6A—C10A—C10B—C4A	−2.8 (3)	C25—C26—C27—C28	−0.1 (3)
C14—O4—C13—O2	−62.31 (16)	C26—C27—C28—C23	−0.3 (3)
C14—O4—C13—C17	−177.13 (13)	C24—C23—C28—C27	0.7 (3)
C14—O4—C13—C16	55.67 (17)	C16—C23—C28—C27	176.17 (18)

### Hydrogen-bond geometry (Å, º)

Cg1 is the centroid of the C4A,C5,C6,C6A,C10A,C10B benzene ring.

**Table:**

*D*—H···*A*	*D*—H	H···*A*	*D*···*A*	*D*—H···*A*
C18—H18···O3	0.95	2.36	3.001 (3)	124
C22—H22···O4	0.95	2.32	2.683 (3)	102
C24—H24···O4	0.95	2.44	3.071 (2)	124
C8—H8···*Cg*1^i^	0.95	2.65	3.3134 (19)	128
C15—H15*A*···*Cg*1^ii^	0.99	2.39	3.336 (2)	161

Symmetry codes: (i) *x*+1/2, −*y*−1/2, *z*+1/2; (ii) −*x*, −*y*, −*z*.
